# Exposure levels of dust, endotoxin, and microorganisms in the Danish recycling industry

**DOI:** 10.1093/annweh/wxad025

**Published:** 2023-05-16

**Authors:** Karoline Kærgaard Hansen, Vivi Schlünssen, Karin Broberg, Kirsten Østergaard, Margit W Frederiksen, Anne Mette Madsen, Henrik Albert Kolstad

**Affiliations:** Department of Occupational Medicine, Danish Ramazzini Centre, Aarhus University Hospital, DK-8200 Aarhus N, Denmark; Department of Public Health, Research Unit for Environment Occupation and Health, Danish Ramazzini Centre, Aarhus University, DK-8000 Aarhus C, Denmark; Division of Occupational and Environmental Medicine, Lund University, SE-221 85 Lund, Sweden; Institute of Environmental Medicine, Karolinska Institutet, SE-17177 Stockholm, Sweden; Department of Public Health, Research Unit for Environment Occupation and Health, Danish Ramazzini Centre, Aarhus University, DK-8000 Aarhus C, Denmark; National Research Centre of the Working Environment, DK-2100 Copenhagen Ø, Denmark; National Research Centre of the Working Environment, DK-2100 Copenhagen Ø, Denmark; Department of Occupational Medicine, Danish Ramazzini Centre, Aarhus University Hospital, DK-8200 Aarhus N, Denmark; Institute of Clinical Medicine, Occupational Medicine, Aarhus University, DK-8200 Aarhus N, Denmark

**Keywords:** bacteria, bioaerosol, domestic waste, endotoxin, fungi, inhalable dust, Occupational exposure, organic dust, recycling workers

## Abstract

**Introduction:**

Recycling of domestic waste and a number of employees in the recycling industry is expected to increase. This study aims to quantify current exposure levels of inhalable dust, endotoxin, and microorganisms and to identify determinants of exposure among recycling workers.

**Methods:**

This cross-sectional study included 170 full-shift measurements from 88 production workers and 14 administrative workers from 12 recycling companies in Denmark. The companies recycle domestic waste (sorting, shredding, and extracting materials from waste). We collected inhalable dust with personal samplers that were analysed for endotoxin (*n* = 170) and microorganisms (*n* = 101). Exposure levels of inhalable dust, endotoxin, and microorganisms and potential determinants of exposure were explored by mixed-effects models.

**Results:**

The production workers were 7-fold or higher exposed to inhalable dust, endotoxin, bacteria, and fungi than the administrative workers. Among production workers recycling domestic waste, the geometric mean exposure level was 0.6 mg/m^3^ for inhalable dust, 10.7 endotoxin unit (EU)/m^3^ for endotoxin, 1.6 × 10^4^ colony forming units (CFU)/m³ of bacteria, 4.4 × 10^4^ CFU/m³ of fungi (25 °C), and 1.0 × 10^3^ CFU/m³ of fungi (37 °C). Workers handling paper or cardboard had higher exposure levels than workers handling other waste fractions. The temperature did not affect exposure levels, although there was a tendency toward increased exposure to bacteria and fungi with higher temperatures. For inhalable dust and endotoxin, exposure levels during outdoor work were low compared to indoor work. For bacteria and fungi, indoor ventilation decreased exposure. The work task, waste fraction, temperature, location, mechanical ventilation, and the company size explained around half of the variance of levels of inhalable dust, endotoxin, bacteria, and fungi.

**Conclusion:**

The production workers of the Danish recycling industry participating in this study had higher exposure levels of inhalable dust, endotoxin, bacteria, and fungi than the administrative workers. Exposure levels of inhalable dust and endotoxin among recycling workers in Denmark were generally below established or suggested occupational exposure limits (OEL). However, 43% to 58% of the individual measurements of bacteria and fungi were above the suggested OEL. The waste fraction was the most influential determinant for exposure, and the highest exposure levels were seen during handling paper or cardboard. Future studies should examine the relationship between exposure levels and health effects among workers recycling domestic waste.

**What’s Important About this Paper**

This study showed that exposure levels to inhalable dust and endotoxin among Danish recycling workers were generally low, however, 43% to 58% of the measurements were above suggested occupational exposure limits for microorganisms. The waste fraction was the most influential determinant for exposure, and the highest exposure levels were seen during the recycling of paper or cardboard. These exposures are a concern as domestic waste recycling and employment in that sector are increasing.

## Introduction

Several of the United Nations (UN) 17 Sustainable Development Goals focus on the environment, including goal 12: responsible consumption and production, which obligates UN member states to ensure sustainable consumption and forms of production (United Nations 2015). From 1992 to 2018, China imported a cumulative 45% of all plastic waste globally, of which the majority came from high-income countries (Brooks et al. 2018), but in 2018, China introduced an import ban on plastic waste (Brooks et al. 2018). High-income countries are now investing in the recycling of domestic waste (waste from the household) (Hopewell et al. 2009; European Commission 2018; McKinsey&Company and Innovation Fund Denmark 2019). Also, one of the goals of the European Union’s “European Green Deal” is to improve the recycling of materials (European Commission 2019).

The European Union (EU) generated 2151 million tonnes of waste in 2020, of which 39.2% was recycled (Eurostat 2021). The amount of domestic waste and the proportion recycled are expected to increase (Eurostat 2021). Despite the increasing automation of recycling of domestic waste, the number of employees in the Danish waste and recycling industry increased by 10% from 2008 to 2020 (Danmarks Statistik 2020), and it is expected that more people will be employed in the recycling industry in the future. In Denmark, each municipality has the main responsibility for the sorting and collection of domestic waste according to the National Plan for Prevention and Management of Waste 2020 to 2032 (Legal Information 2021; Ministry of Environment of Denmark 2021).

High exposure levels of dust, endotoxin, bacteria, and fungi have previously been observed when collecting (Wouters et al. 2002; Madsen et al. 2021; Moller et al. 2022) and sorting (Park et al. 2011; Pinto et al. 2015; Schlosser et al. 2015; Cerna et al. 2017; Kozajda et al. 2017; Baghani et al. 2022) domestic waste. In a Danish context, recent studies have investigated exposures during the collection of domestic waste (Madsen et al., 2016, 2019, 2020), and recycling of domestic biowaste (biodegradable garden, park, food and kitchen waste (Legal Information 2021)) (Rasmussen et al. 2021). Exposure to dust, endotoxin, bacteria, and fungi during the recycling of domestic waste was investigated in Denmark in the early nineties (Malmros et al. 1992; Sigsgaard et al. 1997), but since then, the Danish waste management and types of waste have changed from earlier dumping on landfills to today’s separation of up to 10 waste fractions that are mechanically or manually separated after collection (Fischer and Mckinnon 2012, Malmros 1997; European Environment Agency 2013; OECD 2019). Therefore, there is a need for studies characterising the current exposure levels of dust, endotoxin, bacteria, and fungi as well as determinants for these exposures in the Danish recycling industry to inform efficient preventive measures.

Previous studies found that work task, waste fraction, indoor versus outdoor location, ventilation, season, and outdoor temperature determined exposure levels of inhalable dust, endotoxin, and microorganisms (Nielsen et al. 1997; Wouters et al. 2006; Park et al. 2011; Madsen et al. 2021). Poulsen et al. (1995) found that company size may influence working conditions and hence the level of exposure among waste collectors.

The occupational exposure limit (OEL) for total dust in Denmark is 3 mg/m^3^, approximately equivalent to 4.5 mg/m^3^ of inhalable dust (Poulsen et al. 1995; Basinas et al. 2016; Legal Information 2019). To date, no OEL is available for airborne endotoxin, neither in Denmark nor elsewhere, but the Dutch Expert Committee on Occupational Safety (DECOS) has suggested a health-based OEL for long-term occupational exposure to airborne endotoxin of 90 EU/m^3^ based on no observed effect level (NOEL) for respiratory symptoms (DECOS 2010). No OEL has been set for airborne microorganisms, but Malmros et al. (1992) and Heida et al. (1995) suggested a health-based OEL for airborne bacteria of 10^4^ CFU/m^3^ (Malmros et al. 1992; Heida et al. 1995). Dutkiewicz (1997) and Gorny and Dutkiewicz (2002) suggested a health-based OEL about work-related respiratory disorders for fungi able to grow at 25 °C (typical room temperature) of 5 × 10^4^ CFU/m^3^ (Dutkiewicz, 1997; Gorny and Dutkiewicz, 2002). No OEL has been proposed for fungi that can grow at 37 °C (the human body temperature), but many of these fungal species related to work with waste are human pathogens (Rasmussen et al. 2021; Madsen et al. 2022). Bacteria and fungi present in organic dust are typically noninfectious. However, they can have adverse effects on the respiratory tract such as mucous membrane irritation, organic dust toxic syndrome, allergic alveolitis and asthma (Dutkiewicz 1997).

The present study aims to document current exposure levels of inhalable dust, endotoxin, and microorganisms and to identify determinants of these exposures among Danish production workers who recycle domestic waste.

## Materials and Methods

### Study population

This study investigated production workers in the recycling industry, who recycled domestic waste (sorted, shredded, and extracted materials from waste). Based on the Danish National Waste Register, we contacted 26 large and small, private and public companies in Jutland and Funen in Denmark. Inclusion criteria for the companies were the employment of day-shift workers that conducted recycling of domestic waste. Information meetings with the management and the employees were held and all received flyers describing the project. If more than 9 production workers (the number of workers possible to sample per day) agreed to participate per company, participants were selected to represent as many different work tasks as possible. The industry is male-dominated, thus only men were included. At least 1 administrative worker per company matched by gender was recruited.

The study was registered at the repository of the Central Denmark Region (1-16-02-715-20), and the Central Denmark Region Ethical Committee approved the study (1-10-72-322-20). All participants gave informed consent and could withdraw from the study at any time.

### Data collection

Data collection was performed on Tuesdays, Wednesdays, or Thursdays with each company visited twice, once in April through June 2021 and once in August through October 2021. Repeated measurements were performed to take season and day-to-day variations in exposure levels into account. The median temperature at noon on the measurement day was obtained at the municipality level from the Danish Meteorological Institute (Danish Meteorological Institute 2022). Company size was based on data from the Danish Central Business Register.

#### Inhalable dust and endotoxin:

We collected inhalable dust on all participants with personal conductive plastic inhalable conical sampler (CIS) cartridges (JS Holdings, Stevenage, United Kingdom) connected to an SKC AirChek XR5000 portable pump (SKC Inc., Eighty-Four, PA) with a flow rate of 3.5 l/min flow. During sampling, the flow was checked with the SKC chek-mate Flowmeter (SKC Inc., Eighty-Four, PA) every 2 to 3 h and during breaks. The inhalable dust was collected on 37-mm glass fibre with a 1.6 µm pore diameter (GFA) filter (Whatman International Ltd., Maidstone, United Kingdom). The cassette was placed on the upper part of the participant’s chest to measure the breathing zone and thus outside any respirator used. We measured a full working day; however, pumps were turned off during breaks lasting more than 15 min. Inhalable dust was determined gravimetrically, and the filters were stored for a minimum of 24 h at 22 °C and 45% relative humidity before weighing using a Mettler UMT2 analytical scale (Mettler-Toledo Ltd., Greifensee, Switzerland) with 0.1-mg precision. For each visit, one field blank was included (*n* = 24). The lower limit of detection (LOD) for inhalable dust was calculated as three times the SD of the mean of the field blanks, corresponding to a LOD of 14 µg/m^3^.

Endotoxin was extracted from the filters, and exposure levels were determined by the Limulus Amebocyte Lysate (LAL) test (Kinetic-QCL 50-650U kit, Lonza, Walkersville, Maryland, United States of America). The extraction of the samples was performed in 5 ml of pyrogen-free water with 0.05% (v/v) Tween-20 (Spaan et al. 2008). The samples were initially shaken for 60 min on a Multi Reax digital shaker (Heidolph Instruments GmbH, Schwabach, Germany) and then centrifuged for 15 min at 1000 × *g*. Subsequently, 1 ml of the supernatant was removed, aliquoted in four 0.1 ml portions, and stored at minus 80 °C. A standard curve obtained from an *Escherichia coli* O55:B5 reference endotoxin was used to determine the exposure levels quantified as endotoxin units (EU) (Basinas et al. 2012). The analytical level of quantification (LOQ) for endotoxin was 0.05 EU/m^3^.

#### Microorganisms:

Microorganisms were collected in parallel with inhalable dust, but only from participants working with waste fractions that had been in contact with organic material (primarily fractions that may contain food scraps). The microorganisms were collected on 37 mm polycarbonate filters using CIS cartridges (JS Holdings, Stevenage, United Kingdom) connected to an SKC AirChek XR5000 portable pump (SKC Inc., Eighty-Four, PA). After sampling, the filters were placed in extraction solution (MilliQ water with 0.85% NaCl and 0.05% Tween80) with 33% glycerol and frozen at –80 °C. The dust from the filters was extracted by orbital shaking at 500 rpm for 15 min at room temperature. Dust suspensions were plated on Dichloran Glycerol agar (DG-18 agar; Thermo Fisher Scientific Oxoid, Basingstoke, United Kingdom) in different dilutions and incubated at 25 °C and 37 °C (to encourage the growth of human pathogens) for 7 and 3 days, respectively, for identification and quantification of fungi. Dust suspensions were also plated in different dilutions on Nutrient agar (NA; Thermo Fisher Scientific Oxoid, Basingstoke, United Kingdom) plates with actidione (cycloheximide; 50 mg/l; Serva, Germany) and incubated at 25 °C for 7 days for identification and quantification of fungi and bacteria, respectively. The plates were inspected every day. Furthermore, dust suspensions were plated on Fastidious Anaerobe Agar (FAA) and incubated anaerobically for 40 h at 37 °C. All visible bacterial colonies on plates with 20 to 150 colonies and all visible fungal colonies on plates with 5 to 40 colonies were counted (Madsen et al., 2016; Rasmussen et al., 2021). The LOD was 42 CFU fungi or bacteria/m^3^.

#### Interviews and observations at the workplaces:

During each measurement day, we collected by a structured interview for each participant the following potential exposure determinants: primary work task, waste fractions handled, indoor or outdoor location, filtered ventilation in the vehicle or indoor mechanical ventilation, and use of personal protective equipment (PPE). If there was a discrepancy between what we observed and what the participants reported, the observations overruled self-reports.

### Classification of potential exposure determinants

Primary work task was defined as the main task the worker performed during the measurement day and was categorized into sorting (manual sorting), machine operation, driving (forklift, crane, excavator), or cleaning and maintenance. Some participants (*n* = 14) reported that they spent an equal amount of time on two or more tasks. Based on the expert assessment, the task with expected higher exposure was then selected with the following ranking: 1 sorting, 2 cleanings and maintenance, 3 machine operation, and 4 driving. Waste was categorized into electronics and hazardous (classified as hazardous waste according to Executive Order on Waste (Legal Information, 2021), i.e. light bulbs, paint, and spray cans), metal, plastic, biowaste, paper or cardboard, metal/plastic/glass, or mixed (more than one fraction of waste fraction, including working at a recycling station). We cross-tabulated all potential exposure determinants pairwise checking for collinearity. Location and mechanical ventilation were highly correlated as the latter was only established indoors. We, therefore, combined them into one variable classified as room/vehicle with mechanical ventilation, room/vehicle without mechanical ventilation, or outdoors. Season and outside temperature were strongly correlated, and thus only outside temperature was included and categorized into ≤10 ºC, 10 to 20 ºC, and >20 ºC. No strong correlation was observed for the remainder of all potential exposure determinants. Company size was categorized according to the number of employees into <50, 51 to 99, or >99.

### Statistical analysis

Inhalable dust, endotoxin and microorganisms were log-normally distributed. Hence, statistical analyses were performed using log-transformed values. All left-censored values (values below LOD/LOQ) were given a value corresponding to LOD/2 (Whitcomb and Schisterman 2008).

For each potential exposure determinant, exposure levels were expressed as arithmetic means (AM) and geometric means (GM). The analyses were conducted using mixed effect models with workers as random effects. In the first step, we assessed the SD (within the worker, between workers, and geometric standard deviation, GSD), as $\exp\left(\sigma_{Within}\right)$, $exp\left(\sigma_{Between}\right)$ and $exp\left(\sqrt{\sigma_{Within}^{2}+\sigma_{Between}^{2}}\right)$, where σ_Between_ and σ_Within_ are the between and within worker SD.

In the next step, we compared production workers by work task and waste fraction with administrative workers (reference category). Unadjusted β-coefficients are presented as exp(β) expressing the relative difference in mean exposure compared with the reference with 95% CI.

In the third step, we restricted analyses to production workers as we aimed to identify their determinants of exposure. The mixed models included all potential exposure determinants of inhalable dust, endotoxin, bacteria, and fungi as fixed effects. β-coefficients are reported as exp(β) expressing the mutually adjusted relative difference in mean exposure compared with the reference with 95% CI. The potential exposure determinant with the highest number of samples was a priori selected as the reference group, except for temperature and company size where the lowest value was serving as a natural low-level reference.

Percent of explained variability was calculated as 1-(ratio of corresponding variance from the model with and without fixed effects on the original scale) × 100.

All analyses were carried out using Stata, version 17.

## Results

### Study population

In total, 12 companies participated (participation rate of 46 %). We performed 172 measurements of inhalable dust and endotoxin on 102 participants. One participant dropped out after 1 h, and the sample was discarded. One participant reported that he changed work tasks to exaggerate dust levels, and this sample was also discarded, leaving 170 measurements from 101 participants for further analysis. The median sampling time was 363 min, with an interquartile range (IQR) of 314 to 392 min. The median duration between the 2 measurement days was 106 days, IQR 98 to 119 days.

Among the production workers, 12.1% used filtering face piece-2 or -3 (FFP2 or FFP3) respirators, surgical masks, or air-supplied respirators, 77.8% used plastic apron, gloves, visors or safety goggles, and 10.1% did not use any kind of PPE during the day of the measurements.

For the analysis of bacteria and fungi, we included 60 participants (102 measurements), out of the 102 participants that handled waste fractions and a priori had reported contact with organic material (representing 7 of the 12 companies). One participant dropped out after 1 h, and the sample was discarded, leaving 101 measurements from 59 participants for further analysis. The median sampling time was 365 min, IQR 332 to 395 min and the median duration between the two measurements was 106 days, IQR 106 to 121 days. Among the production workers, 12.8% used FFP2/FFP3 mask, surgical mask, or air-supplied respirator, 76.7% used plastic aprons, gloves, visors, or safety goggles, and 10.5% did not use any kind of PPE during the day of the measurements.

No measurements of endotoxin, 3 of inhalable dust, 3 of fungi (37 ºC), and 1 of bacteria were below LOD/LOQ.

### Exposure levels of inhalable dust, endotoxin, bacteria, and fungi

The GM exposure level among production workers was 0.6 mg/m^3^ for inhalable dust (Table 1), 10.7 EU/m^3^ for endotoxin (Table 1), 1.6 × 10^4^ CFU/m³ for bacteria (Table 2), 4.4 × 10^4^ CFU/m³ for fungi (25 °C) (Table 2), and 1.0 × 10^3^ CFU/m³ for fungi (37 °C) (Table 2). Exposure levels were substantially lower among administrative workers (0.0 mg/m^3^, 1.3 EU/m^3^, 2.2 × 10^3^ CFU/m³, 5.5 × 10^2^ CFU/m³, 1.0 × 10^2^ CFU/m³, respectively).

**Table 1.** Exposure levels of inhalable dust and endotoxin among recycling workers.

	Persons	Samples	Inhalable dust mg/m³						Endotoxin EU^a^/m³					
Characteristics	*K*	*N*	AM^a^	>OEL^a^ (%)	GM^a^	GSD^a^	BW^a^	WW^a^	AM^a^	>OEL^a^ (%)	GM^a^	GSD^a^	BW^a^	WW^a^
Production workers	88	149	1.3	9 (6)	0.6	3.7	2.9	2.1	34.6	12 (8)	10.7	4.2	3.0	2.5
Work task^b^														
Sorting	46	78	1.3	4 (5)	0.6	4.1	3.1	2.2	46.7	9 (12)	11.7	4.8	3.3	2.7
Machine operation	19	28	1.2	3 (11)	0.5	3.2	2.8	1.8	17.6	1 (4)	8.6	3.6	2.6	2.4
Driving	25	36	0.95	1 (3)	0.5	3.0	2.0	2.3	22.9	1 (3)	11.1	3.4	2.4	2.4
Cleaning and maintenance	5	7	4.1	1 (14)	1.5	4.0	3.4	2.0	34.9	1 (14)	12.7	3.5	2.5	2.3
Waste fraction^b^														
Paper or cardboard	17	28	2.1	3 (11)	1.4	2.8	1.9	2.2	98.5	9 (32)	44.3	4.2	2.6	2.9
Metal	15	21	2.9	4 (19)	1.1	4.8	4.3	1.8	31.3	2 (10)	13.2	3.9	2.6	2.7
Plastic	11	17	1.0	1 (6)	0.4	3.2	2.8	1.6	7.2	0 (0)	4.2	2.6	2.1	1.8
Biowaste	6	10	0.4	0 (0)	0.3	1.5	1.3	1.3	26.9	0 (0)	16.7	2.1	1.0	2.1
Electronics and hazardous^c^	14	27	0.7	0 (0)	0.4	2.9	1.9	2.3	4.5	0 (0)	3.2	2.2	1.6	1.8
Metal/plastic/glass	13	22	0.4	0 (0)	0.2	3.2	1.6	2.9	38.4	1 (5)	12.4	2.7	1.0	2.7
Mixed^d^	16	24	1.1	1 (4)	0.5	3.1	2.4	2.1	20.7	0 (0)	9.4	3.4	1.7	3.0
Temperature														
<10 ºC ^e^	23	23	1.0	1 (4)	0.5	3.2	–	–	29.4	0 (0)	20.8	2.3	-	-
10 to 20 ºC	83	111	1.3	8 (7)	0.5	4.0	3.3	2.1	33.6	11 (10)	9.3	4.2	3.1	2.4
>20 ºC ^e^	15	15	0.8	0 (0)	0.6	2.0	–	–	45.2	1 (7)	6.2	4.5	-	-
Location and ventilation^b^														
Room/vehicle ventilation (indoor)	55	86	1.7	8 (9)	0.7	4.3	3.3	2.3	48.7	11 (13)	12.2	5.1	3.7	2.6
No room/vehicle ventilation (indoor)	22	30	0.9	1 (3)	0.5	2.5	2.0	1.8	19.4	1 (3)	9.9	3.2	2.4	2.2
Outdoor	22	33	0.6	0 (0)	0.4	2.5	1.9	1.9	10.9	0 (0)	6.4	2.5	1.0	2.5
Company size^f^														
<50	29	48	0.9	1 (2)	0.4	3.0	2.4	2.0	17.1	2 (4)	7.2	3.2	2.4	2.1
50 to 99	34	58	1.6	4 (7)	0.6	3.7	2.8	2.2	20.2	2 (3)	7.9	3.6	2.3	2.6
>99	25	43	1.5	4 (9)	0.7	4.4	3.4	2.2	78.5	8 (19)	24.4	4.7	3.1	2.9
Administrative workers	14	21	0.1	0 (0)	0.0	2.1	1.2	2.0	1.8	0 (0)	1.3	2.3	1.4	2.1

^a^Endotoxin unit (EU), arithmetic mean (AM), occupational exposure limit (OEL), geometric mean (GM), geometric standard deviation (GSD), between worker (BW) ands within worker (WW).

^b^Primary. For repeated measurements, a person can appear in 2 categories.

^c^Classified as hazardous waste, e.g. light bulbs, paint, and spray cans.

^d^More than 1 fraction of waste fraction, including working at a recycling station.

^e^No repeated measurements, therefore only a geometric standard deviation.

^f^Number of employees.

**Table 2.** Exposure levels of bacteria and fungi among recycling workers.

	Persons	Samples	Bacteria CFU^a^/m³						Fungi CFU^a^/m³, 25 °C						Fungi CFU^a^/m³, 37 °C				
Characteristics	*K*	*N*	AM^a^	>OEL^a^ (%)	GM^a^	GSD^a^	BW^a^	WW^a^	AM^a^	>OEL^a^ (%)	GM^a^	GSD^a^	BW^a^	WW^a^	AM^a^	GM^a^	GSD^a^	BW^a^	WW^a^
Production workers	49	86	5.0 × 10^4^	50 (58)	1.6 × 10^4^	4.5	2.7	3.2	8.4 × 10^5^	37 (43)	4.4 × 10^4^	8.7	5.9	3.4	3.8 × 10^3^	1.0 × 10^3^	5.1	2.6	3.7
Work task^b^																			
Sorting	30	49	5.5 × 10^4^	30 (61)	1.8 × 10^4^	4.6	3.4	2.5	1.3 × 10^6^	30 (61)	7.7 × 10^4^	11.1	6.6	4.5	5.5 × 10^3^	1.3 × 10^3^	5.9	3.8	3.2
Machine operation	7	10	5.3 × 10^4^	5 (50)	1.6 × 10^4^	4.0	1.7	3.6	1.1 × 10^5^	4 (40)	3.4 × 10^4^	4.2	3.2	2.4	3.1 × 10^3^	2.1 × 10^3^	2.5	2.5	1.1
Driving	17	25	3.9 × 10^4^	13 (52)	1.3 × 10^4^	4.6	2.2	3.7	2.4 × 10^4^	2 (8)	1.3 × 10^4^	3.0	2.0	2.4	1.1 × 10^3^	5.4 × 10^2^	3.8	1.0	3.8
Cleaning and maintenance^c^	1	2	1.3 × 10^4^	2 (100)	1.3 × 10^4^	1.1	–	–	4.1 × 10^4^	1 (50)	2.2 × 10^4^	3.4	–	–	3.5 × 10^2^	2.3 × 10^2^	2.7	–	–
Waste fraction^b^																			
Paper or cardboard	17	28	9.8 × 10^4^	23 (82)	4.3 × 10^4^	4.5	3.0	2.8	1.2 × 10^5^	12 (43)	3.3 × 10^4^	4.1	2.4	3.1	6.1 × 10^3^	2.4 × 10^3^	4.1	2.8	2.5
Metal^c^	1	2	4.8 × 10^3^	0 (0)	2.9 × 10^4^	3.0	–	–	1.3 × 10^4^	0 (0)	7.6 × 10^3^	3.1	–	–	4.4 × 10^2^	1.4 × 10^2^	6.0	–	–
Biowaste	6	10	8.5 × 10^4^	5 (50)	2.1 × 10^4^	6.6	1.0	6.6	4.3 × 10^4^	3 (30)	3.2 × 10^4^	2.2	1.0	2.2	1.9 × 10^3^	9.9 × 10^2^	3.3	1.2	3.3
Metal/plastic/glass	13	22	1.5 × 10^4^	12 (55)	9.8 × 10^3^	2.7	1.0	2.7	3.0 × 10^6^	19 (86)	4.4 × 10^5^	9.6	5.5	4.4	5.4 × 10^3^	1.4 × 10^3^	5.2	2.7	3.8
Mixed^d^	16	24	1.4 × 10^4^	10 (42)	8.5 × 10^3^	2.7	1.6	2.3	1.9 × 10^4^	3 (13)	8.4 × 10^3^	3.3	1.1	3.3	9.4 × 10^2^	3.9 × 10^2^	3.7	1.3	3.6
Temperature																			
<10 ºC ^e^	20	20	3.7 × 10^4^	9 (45)	8.8 × 10^3^	4.6	–	–	5.2 × 10^4^	7 (35)	1.9 × 10^4^	4.8	–	–	1.5 × 10^3^	4.8 × 10^2^	4.6	–	–
10 to 20 ºC	45	62	5.4 × 10^4^	37 (60)	1.8 × 10^4^	4.4	3.1	2.6	9.9 × 10^5^	26 (42)	5.1 × 10^4^	9.3	6.3	3.5	3.4 × 10^3^	1.1 × 10^3^	4.4	2.7	3.0
>20 ºC ^e^	4	4	2.4 × 10^4^	4 (100)	2.1 × 10^4^	1.7	–	–	6.4 × 10^5^	4 (100)	6.2 × 10^5^	1.3	–	–	2.1 × 10^4^	1.4 × 10^4^	2.3	–	–
Location and ventilation^b^																			
Room/vehicle ventilation (indoor)	25	41	7.0 × 10^4^	28 (68)	2.3 × 10^4^	4.8	3.5	2.6	1.6 × 10^6^	25 (61)	9.7 × 10^4^	11.2	7.1	4.1	6.0 × 10^3^	1.5 × 10^3^	5.3	2.9	3.6
No room/vehicle ventilation (indoor)	12	20	3.7 × 10^4^	13 (65)	1.5 × 10^4^	4.1	1.0	4.1	1.3 × 10^5^	9 (45)	4.4 × 10^4^	4.0	3.2	2.1	3.1 × 10^3^	1.7 × 10^3^	4.0	1.8	3.5
Outdoor	16	25	2.6 × 10^4^	9 (36)	8.7 × 10^3^	3.5	2.4	2.5	2.2 × 10^4^	3 (12)	9.9 × 10^3^	3.6	1.0	3.6	6.9 × 10^2^	3.7 × 10^2^	3.5	1.4	3.4
Company size^f^																			
<50	14	25	2.7 × 10^4^	12 (48)	9.8 × 10^3^	3.7	1.8	3.3	9.9 × 10^4^	7 (28)	1.9 × 10^4^	5.7	4.4	2.5	1.8 × 10^3^	7.1 × 10^2^	4.8	3.1	3.0
50 to 99	13	23	3.6 × 10^4^	11 (48)	1.2 × 10^4^	3.8	2.3	2.9	5.1 × 10^4^	9 (39)	2.6 × 10^4^	4.3	1.0	4.3	6.0 × 10^2^	4.4 × 10^2^	2.4	1.0	2.4
>99	22	38	7.4 × 10^4^	27 (71)	2.7 × 10^4^	4.7	2.5	3.4	1.8 × 10^6^	21 (55)	1.1 × 10^5^	12.1	9.6	2.9	7.1 × 10^3^	2.2 × 10^3^	5.4	1.0	5.4
Administrative workers	10	15	6.0 × 10^3^	2 (13)	2.2 × 10^3^	4.0	1.0	4.0	7.6 × 10^2^	0 (0)	5.5 × 10^2^	2.3	1.0	2.3	3.3 × 10^2^	1.0 × 10^2^	4.6	1.0	4.6

^a^Colony forming units (CFU), arithmetic mean (AM), occupational exposure limit (OEL), geometric standard deviation (GSD), geometric mean (GM), between worker (BW) and within worker (WW).

^b^Primary. For repeated measurements, a person can appear in two categories. We only collected microorganisms from participants working with waste fractions that have been in contact with organic material (primarily fractions that used to contain food).

^c^Only 1 person, therefore only a geometric standard deviation.

^d^More than 1 fraction of waste fraction, including working at a recycling station.

^e^No repeated measurements, therefore only a geometric standard deviation.

^f^Number of employees.

By work task, the highest exposure levels of inhalable dust and endotoxin were observed during sorting (0.6 mg/m³ and 11.7 EU/m³), and cleaning and maintenance (1.5 mg/m³ and 12.7 EU/m³). For bacteria, fungi (25 °C), and fungi (37 °C), the highest exposure levels were observed during sorting (1.8 × 10^4^ CFU/m³, 7.7 × 10^4^ CFU/m³, and 1.3 × 10^3^ CFU/m³ respectively), and for fungi (37 °C) also among machine operators (2.1 × 10^3^ CFU/m³).

By waste fraction, the highest exposure levels of inhalable dust, endotoxin, bacteria, and fungi (37 °C) were observed during recycling paper or cardboard (1.4 mg/m³, 44.3 EU/m³, 4.3 × 10^4^ CFU/m^3^, and 2.4 × 10^3^ CFU/m³ respectively). For fungi (25 °C), the highest exposure levels were observed during recycling of metal/plastic/glass (4.4 × 10^5^ CFU/m^3^).

The variation between workers (BW) across all potential exposure determinants varied more for fungi (25 °C) (1.0 to 9.6) than for inhalable dust (1.3 to 4.3), endotoxin (1.0 to 3.7), bacteria (1.0 to 3.5), and fungi (37 °C) (1.0 to 3.8). The variation within workers (WW) across all potential exposure determinants varied more for bacteria (2.3 to 6.6) and fungi (37 °C) (1.1 to 6.0) than for inhalable dust (1.3 to 2.9), endotoxin (1.8 to 3.0), and fungi (25 °C) (2.1 to 4.4).

Among production workers, 6% to 58% of the measurements for inhalable dust, endotoxin, bacteria and fungi (25 °C) were above established or suggested OEL (6%, 8%, 58%, and 43%, respectively).

### Determinants of inhalable dust, endotoxin, bacteria, and fungi

Production workers were at least 7-fold higher exposed to inhalable dust, endotoxin, bacteria, and fungi compared with administrative workers (Table 3). Workers cleaning and maintaining had a 25-fold higher exposure to inhalable dust mg/m^3^ and a 10-fold higher exposure to endotoxin EU/m^3^ (exp(β) = 25.0, 95% CI: 7.0;78.9, and exp(β) = 10.0, 95% CI: 2.8;35.6) than the administrative workers. Workers sorting waste had an 8-fold higher exposure to bacteria CFU/m^3^ and a 137-fold higher exposure to fungi (25 °C) CFU/m^3^ (exp(β) = 8.6, 95% CI: 3.4;21.8, and exp(β) = 137.7, 95% CI: 39.9;475.3) compared with the administrative workers. The highest relative difference in exposure levels of inhalable dust, endotoxin, bacteria and fungi (37 °C) was observed among workers handling paper and cardboard compared with administrative workers.

**Table 3.** Determinants of exposure to inhalable dust (mg/m^3^), endotoxin (EU/m^3^) (endotoxin unit, EU), bacteria (CFU/m^3^) (colony forming units, CFU), and fungi (CFU/m^3^) among recycling workers presented as unadjusted β-coefficients (Exp(β)) expressing the relative difference in mean exposure compared with the reference with 95% CI.

	Inhalable dust^a^			Endotoxin^a^			Bacteria^a^			Fungi^a^, 25 °C			Fungi^a^, 37 °C		
	Exp(β)	95% CI	*P*-value	Exp(β)	95% CI	*P*-value	Exp(β)	95% CI	P-value	Exp(β)	95% CI	P-value	Exp(β)	95% CI	P-value
Administrative worker	Reference	Reference	Reference	Reference	Reference	Reference	Reference	Reference	Reference	Reference	Reference	Reference	Reference	Reference	Reference
Production worker	11.9	6.2;22.7	<0.001	7.6	3.7;15.4	<0.001	7.6	3.1;18.4	<0.001	82.2	22.6;298.7	<0.001	10.7	4.2;27.3	<0.001
Work task^c^															
Sorting	11.9	6.0;23.6	<0.001	8.3	3.9;17.5	<0.001	8.6	3.4;21.8	<0.001	137.7	39.9;475.3	<0.001	13.8	5.5;34.4	<0.001
Machine operation	10.2	4.7;22.3	<0.001	5.8	2.4;13.7	<0.001	6.4	1.8;22.6	0.004	59.3	11.7;300.4	<0.001	20.3	5.8;71.5	<0.001
Driving	11.6	5.6;23.8	<0.001	7.5	3.4;16.7	<0.001	6.4	2.3;17.7	<0.001	32.5	8.5;124.7	<0.001	5.4	2.0;14.9	0.001
Cleaning and maintenance	25.0^b^	7.9;78.9^b^	<0.001^b^	10.0^b^	2.8;35.6^b^	<0.001^b^	6.1^b^	0.5;69.9^b^	0.1^b^	41.6^b^	1.4;1235.6^b^	0.031^b^	2.3^b^	0.2;23.8^b^	0. 5^b^
Waste fraction^c^															
Paper/cardboard	25.4	12.5;51.7	<0.001	31.3	15.8;62.0	<0.001	19.3	8.3;44.8	<0.001	58.1	21.0;160.3	<0.001	23.8	9.2;61.7	<0.001
Metal	22.4	10.6;47.6	<0.001	10.1	4.9;20.8	<0.001	1.3^b^	0.2;9.9^b^	0.8^b^	14.2^b^	1.2;170.8^b^	0.037^b^	1.5^b^	0.1;14.1^b^	0.7^b^
Plastic	10.1	4.6;22.1	<0.001	3.2	1.5;6.9	0.003	-	-	-	-	-	-	-	-	-
Biowaste	7.2	2.7;18.9	<0.001	13.2	5.3;33.1	<0.001	10.0	3.4;29.2	<0.001	62.1	16.8;229.4	<0.001	9.9	2.9;33.4	<0.001
Electronics and hazardous^d^	9.6	4.6;22.2	<0.001	2.5	1.2;4.9	0.012	-	-	-	-	-	-	-	-	-
Metal/plastic/glass	4.6	2.1;9.9	<0.001	9.5	4.6;19.6	<0.001	4.6	1.9;11.0	0.001	746.7	256.5;2173.8	<0.001	14.1	5.2;38.3	<0.001
Mixed^e^	12.3	5.9;25.4	<0.001	7.7	3.8;15.6	<0.001	3.9	1.7;9.4	0.002	16.8	6.0;47.4	<0.001	3.9	1.5;10.3	0.006

^a^Unadjusted model including the random effect of worker.

^b^Less than 10 observations. The number of participants and samples are displayed in Table 1 and Table 2.

^c^Primary. For repeated measurements, a person can appear in two categories. We only collected microorganisms from participants working with waste fractions that have been in contact with organic material (primarily fractions that used to contain food).

^d^Classified as hazardous waste, e.g. light bulbs, paint, and spray cans.

^e^More than one fraction of waste fraction, including working at a recycling station.

When mutually adjusting for all potential exposure determinants among the production workers, work tasks did not determine exposure levels of inhalable dust, endotoxin, bacteria, or fungi (Table 4). However, waste fraction did determine exposure levels of inhalable dust, endotoxin, bacteria, and fungi; workers handling paper or cardboard were more exposed than workers handling other waste fractions. Handling metal/plastic/glass resulted in 90% lower mean exposure to inhalable dust (exp(β) = 0.1, 95% CI: 0.048;0.2), and handling electronics and hazardous waste resulted in 95.4% lower mean exposure to endotoxin (exp(β) = 0.046, 95% CI: 0.02;0.1). Handling metal/plastic/glass or mixed fractions compared with handling paper or cardboard decreased exposure levels of bacteria and fungi (37 °C). Handling metal/plastic/glass resulted in 80% lower mean exposure to bacteria (exp(β) = 0.2, 95% CI: 0.1;0.4), and handling mixed fractions resulted in 80% lower mean exposure to fungi (37 °C) (exp(β) = 0.2, 95% CI: 0.1;0.6). In contrast, handling metal/plastic/glass on average increased exposure levels to fungi (25 °C), by 15.6 times (exp(β) = 15.6, 95% CI: 5.9;41.2).

**Table 4.** Determinants of exposure to inhalable dust (mg/m^3^), endotoxin (EU/m^3^) (endotoxin unit, EU), bacteria (CFU/m^3^) (colony forming units, CFU), and fungi (CFU/m^3^) among recycling workers presented as mutually adjusted β-coefficients (Exp(β)) expressing the relative difference in mean exposure compared with the reference with 95% CI.

	Inhalable dust^a^			Endotoxin^a^			Bacteria^a^			Fungi^a^, 25 °C			Fungi^a^, 37 °C		
	Exp(β)	95% CI	*P*-value	Exp(β)	95% CI	*P*-value	Exp(β)	95% CI	*P*-value	Exp(β)	95% CI	P-value	Exp(β)	95% CI	P-value
Intercept	1.9	0.9;4.2	0.1	102.4	48;217	<0.001	2.6 × 10^4^	8.7 × 10^3^ ;7.5 × 10^4^	<0.001	7.8 × 10^3^	2.3 × 10^3^;2.7 × 10^4^	<0.001	747	269-2.0 × 10^3^	<0.001
Work task^c^															
Sorting	Reference	Reference	Reference	Reference	Reference	Reference	Reference	Reference	Reference	Reference	Reference	Reference	Reference	Reference	Reference
Machine operation	0.7	0.4;1.3	0.3	0.6	0.3;1.2	0.1	0.6	0.2;1.7	0.3	0.4	0.1;1.4	0.2	1.6	0.6;4.5	0.4
Driving	0.7	0.4;1.2	0.2	0.6	0.4;1.0	0.059	0.6	0.3;1.1	0.1	0.6	0.2;1.3	0.2	0.5	0.2;0.9	0.025
Cleaning and maintenance	1.3^b^	0.5;3.4^b^	0.5^b^	1.1^b^	0.5;2.4^b^	0.9^b^	1.1^b^	0.2;6.7^b^	0.9^b^	2.6^b^	0.3;22.4^b^	0.4^b^	0.4^b^	0.1;2.5^b^	0.3^b^
Waste fraction^c^															
Paper or cardboard	Reference	Reference	Reference	Reference	Reference	Reference	Reference	Reference	Reference	Reference	Reference	Reference	Reference	Reference	Reference
Metal	1.1	0.5;2.3	0.8	0.4	0.2;0.9	0.018	0.1^b^	0.03;1.2^b^	0.1^b^	0.3^b^	0.03;3.7^b^	0.4^b^	0.1^b^	0.02;1.1^b^	0.06^b^
Plastic	0.6	0.3;1.4	0.2	0.2	0.1;0.4	<0.001	–	–	–	–	–	–	–	-	-
Biowaste	0.3	0.1;0.8	0.018	0.3	0.1;0.9	0.025	0.5	0.1;2.1	0.3	1.2	0.2;6.4	0.8	0.2	0.04;0.6	0.009
Electronics and hazardous^d^	0.2	0.1;0.4	<0.001	0.046	0.024;0.1	<0.001	–	–	–	–	–	–	–	-	-
Metal/plastic/glass	0.1	0.048;0.2	<0.001	0.1	0.1;0.3	<0.001	0.2	0.1;0.4	<0.001	15.6	5.9;41.2	<0.001	0.3	0.1;0.7	0.007
Mixed^e^	0.6	0.3;1.3	0.2	0.2	0.1;0.5	<0.001	0.2	0.1;0.6	0.005	0.2	0.1;0.9	0.030	0.2	0.1;0.6	0.003
Temperature															
<10 ºC	Reference	Reference	Reference	Reference	Reference	Reference	Reference	Reference	Reference	Reference	Reference	Reference	Reference	Reference	Reference
10-20 ºC	0.6	0.4;1.0	0.031	0.3	0.2;0.6	<0.001	1.2	0.6;2.5	0.6	2.1	1.0;4.5	0.1	1.5	0.8;3.0	0.2
>20 ºC	1.1	0.5;2.2	0.8	0.5	0.3;1.1	0.1	1.9 ^b^	0.4;8.4^b^	0.4^b^	1.1^b^	0.2;5.3^b^	0.9^b^	17.0^b^	4.3;67.7^b^	<0.001^b^
Location/ventilation^c^															
Room/vehicle ventilation (indoor)	Reference	Reference	Reference	Reference	Reference	Reference	Reference	Reference	Reference	Reference	Reference	Reference	Reference	Reference	Reference
No room/vehicle ventilation (indoor)	0.9	0.5;1.4	0.5	0.8	0.5;1.3	0.3	1.1	0.5;2.7	0.8	2.2	0.8;6.1	0.1	3.5	1.5;8.3	0.004
Outdoor	0.4	0.2;0.7	0.005	0.4	0.2;0.8	0.011	1.1	0.3;4.2	0.9	2.7	0.6;12.4	0.2	1.9	0.5;6.6	0.3
Company size^f^															
<50	Reference	Reference	Reference	Reference	Reference	Reference	Reference	Reference	Reference	Reference	Reference	Reference	Reference	Reference	Reference
50-99	1.6	0.8;3.2	0.1	1.6	0.9;3.0	0.1	1.9	0.8;4.6	0.2	0.9	0.3;2.5	0.8	1.4	0.6;3.4	0.4
>99	1.9	1.0;3.8	0.1	2.5	1.4;4.6	0.003	2.8	1.2;6.5	0.021	4.4	1.6;12.2	0.004	3.5	1.5;8.2	0.003

^a^Model including the fixed effect of task, waste fraction, location, ventilation, and company size and random effect of worker.

^b^Less than 10 observations. The number of participants and samples are displayed in Tables 1 and 2.

^c^Primary. For repeated measurements, a person can appear in two categories. We only collected microorganisms from participants working with waste fractions that have been in contact with organic material (primarily fractions that used to contain food) .

^d^Classified as hazardous waste, e.g. light bulbs, paint, and spray cans.

^e^More than one fraction of waste fraction, including working at a recycling station.

^f^Number of employees.

The temperature did not influence exposure levels of inhalable dust, endotoxin, bacteria, or fungi, although there was a tendency to an association between higher temperature and exposure levels of bacteria and fungi (37 °C). Working outdoors compared with working indoors with ventilation on average decreased exposure levels of inhalable dust by 60% (exp(β) = 0.4, 95% CI: 0.2;0.7), and endotoxin by 60% (exp(β) = 0.4, 95% CI: 0.2;0.8). For bacteria and fungi, no consistent effect was seen, but working indoors with no room/vehicle ventilation compared with working indoors with ventilation increased exposure to fungi (37 °C) by 3.5 times (exp(β) = 3.5, 95% CI: 1.5;8.3). Company size did not show a consistent association with exposure levels of inhalable dust, endotoxin, bacteria, or fungi.

### Variance components for inhalable dust, endotoxin, bacteria, and fungi

In the naïve models for inhalable dust, endotoxin, bacteria, fungi (25 °C), and fungi (37 °C), the total variance was equally distributed between workers (30.0% to 68.1%), and within-worker (31.9% to 70.0%). Including task, waste fraction, temperature, location, ventilation, and company size as fixed effects explained between 39.1% to 61.7% of the total variance, primarily decreasing the variability between workers (72.7% to 100% explained) (Table 5).

**Table 5.** Variance components (expressed on original log-scale) for inhalable dust, endotoxin, bacteria, and fungi from models excluding and including fixed effects.

	Inhalable dust			Endotoxin			Bacteria			Fungi, 25 °C			Fungi, 37 °C		
	Exc. fixed effects	Inc. fixed effects^a^	% explained^b^	Exc. fixed effects	Inc. fixed effects^a^	% explained^b^	Exc. fixed effects	Inc. fixed effects^a^	% explained^b^	Exc. fixed effects	Inc. fixed effects^a^	% explained^b^	Exc. fixed effects	Inc. fixed effects^a^	% explained^b^
Between-worker variance	1.1	0.3	72.7	1.2	0.1	91.7	1.0	0.0	100.0	3.2	0.3	90.6	0.9	0.1	88.9
Within worker variance	0.6	0.7	–16.7	0.8	0.8	0.0	1.3	1.4	–7.7	1.5	1.5	0.0	1.7	1.2	29.4
Total variance	1.7	1.0	41.2	2.0	0.9	55.5	2.3	1.4	39.1	4.7	1.8	61.7	2.6	1.3	50.0

^a^Model including task, waste fraction, temperature, location/ventilation, and company size as fixed effects.

^b^Percent of explained variability was calculated as 1-(ratio of corresponding variance from model with and without fixed effects on original scale)×100.

## Discussion

### Main results

The production workers of this study were 7-fold or higher exposed to inhalable dust, endotoxin, bacteria, and fungi than administrative workers. This study identified determinants of high exposure levels of inhalable dust, endotoxin, bacteria, and fungi in the current Danish recycling industry. Workers handling paper or cardboard were more exposed than workers handling other waste fractions and in turn higher than administrative workers. The temperature did not consistently influence the exposure levels, although there was a tendency towards higher exposure to bacteria and fungi with higher temperatures. For inhalable dust and endotoxin, exposure levels for outdoor work were low compared to indoor work, whereas for bacteria and fungi, indoor ventilation decreased exposure. The task, waste fraction, temperature, location, mechanical ventilation, and the company explained around half of the variance of inhalable dust, endotoxin, bacteria, and fungi levels.

### Previous studies

Park et al. (2011) found a higher GM of total dust among Korean workers collecting and sorting domestic waste (0.6 mg/m^3^), endotoxin (220 EU/m^3^), bacteria (0.2 × 10^5^ CFU/m³), and partly fungi (0.9 × 10^4^ CFU/m^3^) than we did for the production workers. A reason for the differences could be that they included workers both collecting and sorting waste and not sorting and recycling the waste as in our study. Kozajda et al. found a higher GM exposure to inhalable dust (1.3 mg/m^3^) and endotoxin (205.5 EU/m^3^) at Polish domestic waste sorting plants (Kozajda et al. 2017) compared with our study. This is maybe because Kozajda et al. (2017) only sampled during summer, while we sampled during spring, summer, and autumn. Furthermore, Kozajda et al. used different filter types for sampling than we did. Pinto et al. (2015) at Portuguese glass sorting plants, found similar AM exposure to bacteria as we did (1.6 × 10^4^ CFU/m^3^), but lower exposure to fungi (25 °C) (1.5 × 10^4^ CFU/m^3^) (Pinto et al. 2015). The difference in fungal exposure could be due to that we used personal samples, whereas Pinto et al. (2015) used stationary samples.

In the Danish context, most recent studies investigated waste collectors (Madsen et al. 2016; Moller et al. 2022). Madsen et al. (2016) found a lower GM of bacteria (1.1 × 10^3^ CFU/m^3^) and fungi (5.7 × 10^3^ CFU/m^3^) among Danish domestic waste collectors than we found for waste recycling workers. Madsen et al. (2016) sampled during winter while we did during spring, summer, and autumn. Moller et al. (2022) collected samples during summer and autumn among Danish workers collecting waste and found a lower GM of bacteria (1.64 × 10^3^ CFU/m^3^) and fungi (7.75 × 10^3^ CFU/m^3^ and 471 CFU/m^3^ for incubation at 25 °C and 37 °C, respectively) than we did. Therefore, the differences in exposures could be due to different work tasks as Madsen et al. (2016) and Moller et al. (2022) investigated workers collecting waste, whereas we investigated workers recycling waste.

In a Danish context, only a few studies have investigated exposures to dust, endotoxin, and microorganisms among workers recycling domestic waste. Sigsgaard et al. (1997) found among workers sorting paper a lower AM exposure level of total dust (0.83 mg/m^3^), endotoxin (1.3 ng/m^3^), and total microorganisms (4.7 × 10^3^ CFU/m^3^) than we did. While the workers in Sigsgaard et al. (1997) only worked with sorting paper, the workers in our study also handled cardboard, and they performed other tasks besides sorting (also machine operation and driving). Also, since the early nineties, both waste management and waste fractions have changed. In recent years, Rasmussen et al. (2021) found among workers recycling biowaste a higher GM of endotoxins (28 EU/m^3^), bacteria (7.6 × 10^4^ CFU/m^3^), fungi able to grow at 25 °C (8.4 × 10^4^ CFU/m^3^), and fungi able to grow at 37 °C (5.9 × 10^3^) than we did. A reason for these differences in exposures might be due to waste fraction, as we also found the highest exposure levels among workers handling paper or cardboard, but also high exposure levels of bacteria among workers handling biowaste.

We found that waste fraction was an important determinant of inhalable dust and endotoxin exposure, and to a lesser extent also location and ventilation, which is in accordance with previous studies (Nielsen et al. 1997; Ivens et al. 1999; Wouters et al. 2006; Park et al. 2011). Park et al. (2011) found that endotoxin exposures were higher when the outdoor temperature exceeded 20 °C, and Madsen et al. (2021) found that exposure to endotoxin was associated with higher outdoor temperatures, but we found no consistent effect of temperature on endotoxin exposure. A reason for this could be the shorter storing time of the waste in our study, but we did not have information on this. Also, 78% of the production workers primarily worked indoors, and thus another reason for this could be due to the air change rate, as the air change may be lower when the temperature is lower due to closed windows and doors.

We found a tendency toward higher temperatures being associated with higher exposure to bacteria and fungi, which is in accordance with findings from Madsen et al. (2021) and Pinto et al (2015), who found that exposure to bacteria and fungi was associated with higher outdoor temperatures. Park et al. (2011) found the highest exposure for work processes in which nonseparated waste was sorted into fractions for incineration, recycling, or other uses, but we found no effect on task. A reason for this could be due to nondifferential misclassification, as we only looked at primary work tasks.

### Strengths and limitations

If companies with high dust exposure levels were more reluctant to participate, we may have underestimated the exposure. In Denmark, companies are obligated to comply with the established OEL for total dust. We speculate that larger companies, with a working environment organization and regular dust exposure monitoring, would be more prone to participate in this study if their exposure levels are below OELs. Companies that do not measure exposure will not know their exposure levels and thus are expected to be more representative. We tried to account for such a selection by recruiting both small and large companies. Worker participation was voluntary, and may also cause selection bias, but we tried to account for this selection by selecting the tasks and the measurement days.

No companies recycling domestic plastic waste wanted to participate. We, therefore include 2 companies recycling industrial plastic waste. The processes are the same, but we assume that the plastic fraction from the industry is cleaner, as it has not been in contact with food scraps. We, therefore, assume that the exposure levels of endotoxin found at these companies are lower than those at companies handling domestic plastic waste.

We obtained information on the potential exposure determinants primarily through interviews, but since the workers were unaware of exposure levels, this information is probably not biased (Hardt et al., 2014). Furthermore, if there were discrepancies with our observations, observations overruled self-reports. Recall bias should not be of concern as all the self-reported information is related to the day of the measurement.

Collinearity was of concern in this study as the potential exposure determinants of location and mechanical ventilation were highly correlated as the latter was only established indoors. We, therefore, combined them into 1 variable classified as room/vehicle with mechanical ventilation, room/vehicle without mechanical ventilation, or outdoors.

For the mutually adjusted mixed-effect models of exposure levels of bacteria and fungi, the risk of overfitting may be of concern. An alternative for these analyses was to exclude some of the a priori selected potential exposure determinants. We decided to rather keep all potential exposure determinants in and the potential risk of overfitting rather than risking potential bias by forward or backward procedures (VanderWeele, 2019).

In this study, we had repeated measurements for most workers. This enabled us to assess variance components both within and between workers and to evaluate how much the included fixed effects decreased the variability in exposure. This knowledge is important for the evaluation of preventive measures. Including task, waste fraction, temperature, location and ventilation, and company as fixed effects in our models decreased the variability between workers by 72.7% to 100% depending on the exposure.

### Comparison with existing and suggested occupational exposure limits

The overall geometric mean exposure for all productions workers was 0.6 mg/m^3^ for inhalable dust, 10.7 EU/m^3^ for endotoxin, and 4.4 × 10^4^ CFU/m³ for fungi (25 °C), and thus below the established or suggested OELs of 4.5 mg/m^3^, 90 EU/m^3^ and 5 × 10^4^ CFU/m^3^. However, 10%, 8%, and 43% of the samples exceeded the OELs for inhalable dust, endotoxin, and fungi (25 °C), respectively. The overall GM among production workers sorting and recycling domestic waste was 1.6 × 10^4^ CFU/m³ for bacteria, and thus the mean exposure levels exceeded the suggested OEL for bacteria (10^4^ CFU/m^3^), with 58% above the suggested OEL. No OEL has been proposed for fungi able to grow at 37 °C, but previous measurements in the waste industry have shown that a large fraction of the fungi may be pathogens, and thus of concern (Rasmussen et al. 2021; Madsen et al. 2022).

## Conclusion

The production workers of the Danish recycling industry participating in this study were 7-fold or higher exposed to inhalable dust, endotoxin, bacteria, and fungi than the administrative workers. Exposure levels of inhalable dust and endotoxin were generally below established or suggested occupational exposure limits (OEL). However, 43% to 58% of the individual measurements of bacteria and fungi were above suggested OELs. The highest exposure levels were seen during handling paper or cardboard. The task, waste fraction, temperature, location, mechanical ventilation, and the company explained around half of the variance of levels of inhalable dust, endotoxin, bacteria, and fungi, and underpins the potential for reducing exposure by affecting these potential exposure determinants. Future studies should examine the relationship between exposure levels and health effects among workers recycling domestic waste.

## Data Availability

The data underlying this paper cannot be shared publicly due to the privacy of the individuals and companies who participated in the study, but data can be shared in an anonymized form on reasonable request to the corresponding author.
